# The effect of *Cinnamomum zeylanicum* essential oil on chemical characteristics of Lyoner- type sausage during refrigerated storage

**Published:** 2015-03-15

**Authors:** Majid Aminzare, Javad Aliakbarlu, Hossein Tajik

**Affiliations:** *Department of Food Hygiene and Quality Control, Faculty of Veterinary Medicine, Urmia University, Urmia, Iran.*

**Keywords:** Chemical characteristics, *Cinnamomum zeylanicum*, Essential oil, Lyoner sausage

## Abstract

The effect of *Cinnamomum zeylanicum* essential oil (CZEO) at two concentrations (0.02% and 0.04% v/w) on chemical composition, pH, water activity (aw), lipid oxidation, color stability and sensory characteristics of Lyoner-type sausage stored at 4 ˚C for 40 days was investigated. The moisture content of the control sample was higher (*p *< 0.05) than CZEO incorporated samples, while fat, ash and protein content were not affected by adding essential oil. At days 0 and 40, Lightness (L*) and whiteness index (WI) were significantly decreased and total color difference (ΔE) significantly increased (*p* < 0.05) by adding CZEO. With the exception of first day of storage, redness (a*) and yellowness (b*) were significantly increased and decreased, respectively during the rest of storage (*p* < 0.05). The pH values were not differing between the control samples and samples containing CZEO (*p* > 0.05). The water activity content fell in Lyoners with added CZEO during the storage. Incorporation of CZEO retard lipid oxidation process at the end of storage (*p* < 0.05). Samples containing highest amount of CZEO had higher sensory score compared to control sample. Our results pointed out that CZEO could be used as natural additive for increasing the chemical stability of Lyoner-type sausages.

## Introduction

Meat products are widely consumed foodstuffs. In addition to appreciable sensory aspects, processed meat products are relatively inexpensive compared with traditional fresh meat cuts. Lyoner is a cured, emulsified and stuffed meat product providing inexpensive access to animal proteins, making the minimal recommended protein intake possible.^1^ Plant-based products are outstanding alternatives to chemical preservatives, and their use in foods meets consumer demands for minimally processed natural products while providing some extra benefits to both food and consumer.^2^ There is considerable evidence from epidemiological, clinical and biochemical studies those essential oils (EOs) and extracts confer a strong positive influence on human health by having high antioxidant pro-perties.^3^^,^^4^ The EOs are aromatic oily liquids obtained from different parts of plant (flowers, buds, seeds, leaves, twig bark, herbs, wood, fruits, and roots) which characterized by a strong specific odor. They are formed by aromatic plants as secondary metabolites.^5^ A complex mix face of compounds constitutes essential oils, including terpenes, alcohols, acetones, phenols, acids, aldehydes and esters.^6^

The essential oils have been extensively used in food products, for their antibacterial, antifungal and antioxidant properties as well as food flavoring agents.^7^ The main advantage of EOs is that they can be used in any food and are generally recognized as safe,^8^ providing that their maximum effects are attained with minimal change in the organoleptic properties of the food.^9^ The use of natural additives has attracted attention, reportedly natural compounds have antioxidant effects similar to or better than those of synthetic preservatives.^10^ The EOs contain many phytochemicals including phenolic compounds like flavonoids, which are sources of natural antioxidants. Their antioxidant activity depends upon their ability to interact with free radicals.^11^ In addition to antioxidant activity, herbal compounds have antimicrobial, anti-inflammatory, anti-mutagenic and anti-cancer activities, which have positive effects on functionality of foods against diseases.^12^^-^^15^ Cinnamon (*Cinnamomum zeylanicum *or *C. verum*), rich in essential oils (EO), belongs to *Lauraceae *family and usually grows in South and South-East Asia.^16^ The inner bark of the tree has been used in ethno-medicine and flavoring for foods.^5^ The *C. zeylanicum* essential oil (CZEO) contains a distinct antioxidant activity, which is attributed to the presence of phenolic and poly-phenolic compounds.^17^^,^^18^ The CZEO acted as a good inhibitor of primary and secondary oxidation products formation in mustard oil at the concentration of 0.02%.^19^ The ability of *C.* *zeylanicum *to retard lipid oxidation is attributable to quenching of reactive oxygen species by its phenolic constituents.^20^ The objective of this study, was to investigate effect of CZEO on the chemical characteristics of Lyoner-type sausage.

## Materials and Methods

**Sausage manufacture. **The Lyoner-type sausages were manufactured in Dara meat products factory located in Shahriar, Iran, according to a traditional formula: 10 kg chicken meat , 13 kg modified deboned chicken meat, 4.6 kg supplement cream including soy, emulsifier, oil, water, 1.6 kg oil, 8.8 kg ice, garlic, white egg, salt, nitrite, dextrose, flour, starch, phosphate, spice, stabilizer, and ascorbic acid. Chicken meat, salt, phosphate and nitrite were placed in a cutter and mixed for 1 min. Fifty percent of the ice and spice were then added and mixed at a high speed. After complete homogenization, the speed of the cutter was reduced. The remaining 50% of ice, starch, ascorbic acid oil were added and mixed until the temperature of the mixture reached 13 ˚C. The total emulsification time was approximately 10 min. The resulting batter was divided into three parts of 5 kg. To obtain different Lyoner samples, various concentrations of CZEO (0.02% and 0.04% v/w) was added to the two divided sample parts. The batters were stuffed into polyamide bags (50 mm caliber; Poshesh Navid, Tehran, Iran) and were cooked by put in cooking room using the following program: 85 ˚C until the temperature of the product reached 73 ˚C (measured by a thermometer inserted into the center of the packed sausage batter). The cooked sausage was cooled in a water bath for 10 min and stored in a controlled chamber at 4 ˚C before analysis at 0, 10, 20, 30 and 40 days. 

**Extraction of CZEO. **Dried aerial parts of *C. zeylanicum* spice were acquired from a local market in Urmia, Iran. The essential oil of *C. zeylanicum* was extracted by hydro-distillation, using a modified Clevenger apparatus method. Plant material was added to water in a 2 L volumetric distillation flask and coupled to the altered Clevenger device and the extraction was performed for 2.5 hr at 100 ˚C. The EO was collected and the remaining water were removed with anhydrous sodium sulfate. The oil was stored at 4 ˚C in glass flasks wrapped in aluminum foil. ^21^

**Identification and quantification of CZEO chemical constituents. **The chemical components of CZEO were identified by gas chromatography with mass spectrometry (GC-MS). A gas chromatograph (Model GC 17A; Shimadzu Corp., Kyoto, Japan) equipped with a mass selective detector (Model QP 5000; Shimadzu Corp., Kyoto, Japan) was operated under the following conditions: fused silica capillary column (30 m, .25 mm) coated with a DB-5 MS stationary phase; ion source temperature of 220 ˚C; column temperature programmed at an initial temperature of 40 ˚C and increased by 3 ˚C per min up to 240 ˚C; helium carrier gas (1 mL per min); initial column pressure of 100 kPa; split ratio of 1:10 and volume injected of 1 µL (1% solution in dichloromethane).

The following conditions were applied for the mass spectrometer (MS): impact energy of 70 eV; decomposition velocity of 1000, analysis interval of 0.50 and fragments of 45 Da and 450 Da decomposed. A mixture of linear hydrocarbons (C9H20; C10H22; C11H24;C24H50; C25H52; C26H54) was injected under identical conditions. The mass spectrum obtained were compared to those of the database (Wiley 229), and the Kovats retention index (KI) calculated for each peak was compared to the values described by Adams.^22^

**Determination of antioxidant activity of EO by 2, 2-diphenyl-1- picrylhydrazyl (DPPH) radical scavenging method. **The hydrogen atom or electron donation ability of the CZEO was measured from the bleaching of purple-colored methanol solution of DPPH.^23^ An amount of 50 μL of CZEO (1, 2 and 4 mg mL^-1^) in methanol was added to 2 mL of a methanol solution of DPPH (24 μg mL^-1^). After shaking, the mixture was incubated at room temperature for 60 min in the dark. Then, the absorbance was measured against a blank at 517 nm using a spectrophotometer (LKB Novaspec II; Pharmacia, Uppsala, Sweden). The radical scavenging activity (RSA) was calculated according to the following equation:

*RSA(%) = (A*_blank_ *− A*_sample_ */A*_blank_ *)×100*

where, *A*_blank_ is the absorbance of the control reaction (containing all reagents except the test compound), and *A*_sample_ is the test compound’s absorbance. BHT (1 mg mL^-1^) was used as a positive control.^24^ All tests were carried out in triplicate and results are reported as mean ± standard deviation of triplicates.

**Measuring composition of sausage samples. **Moisture, ash, protein and fat content of sausage samples were determined by AOAC methods.^25^ Moisture (g water per 100g sample) was determined by drying a 3 g sample at 105 ˚C to constant weight. Ash was performed at 550 ˚C for 2 hr (g ash per 100 g sample). Fat (g fat per 100 g sample) was calculated by weight loss after a six cycle extraction with petroleum ether in a Soxhlet apparatus (Fisher Scientific, Loughborough, UK). Protein (g protein per 100 g sample) was analyzed according to the Kjeldahl method.^25^ A factor of 6.25 was used for the conversion of nitrogen to crude protein.

**Color measurement. **For measurement of color of Lyoner samples, the CIE LAB color space was studied following the procedure of Cassens *et al*.^26^ Color of samples was measured during storage at 4 ˚C after 0, 10, 20, 30 and 40 days by a colorimeter (Model CR-400; Minolta Co., Osaka, Japan) against a white standard. Hunter color scale was used, lightness (L*) and chromaticity parameters redness (a*, ± red–green) and yellowness (b*, ± yellow–blue) were measured. American Meat Science Association guidelines for color measure-ments were followed and spectrally pure glass (Model CRA51; Minolta Co., Osaka, Japan) was put between the samples and the equipment.^27^ Total color difference (ΔE) and whiteness index (WI) were calculated using the following equations:^28^

*ΔE = [(L*_standard_ *-L*_sample_ *)* ^2^ *+ (a*_standard_ *-a*_sample_ *)* ^2^ *+ (b*_standard_ *-b*_sample_ *)* ^2^ *]* ^0.5^

*WI = 100 – [(100 – L)* ^2^ *+ a* ^2^ *+b* ^2^ *]* ^0.5^

**pH and water activity measurement.** The pH was measured by blending a 10 g sample with 90 mL deionized water for 2 min. The pH of the obtained suspension was measured with a Crison pH meter (Model 507; Crison, Barcelona, Spain) equipped with a Crison combination electrode (Cat. no. 52; Crison, Barcelona, Spain).

The water activity (aw) was measured at 25 ˚C using an electric hygrometer (Model TH200; Novasina; Axair Ltd., Pfaeffikon, Switzerland) according to the manufacture instruction.

**Lipid oxidation** **.** Degree of lipid oxidation in different samples was performed using an extraction method of thiobarbituric acid reactive substances (TBARs) method as described previously with minor modifications.^29^ A meat sample (10 g) was homogenized with 35 mL of cold (4 ˚C) extraction solution containing 4% per chloric acid and 1 mL of butylated hydroxy toluene (BHT) (5 g L^-1^) at 13,500 rpm for 1 min. The blended sample was filtered through Whatman filter paper no. 1 (Whatman International Ltd., Maidstone, USA) into a 50-mL falcon tube. The filtrate was adjusted to 50 mL with 4% perchloric acid and 5 mL this mixture was added to 5 mL of thiobarbituric acid (TBA) solution (0.02 mol L^-1^). The mixture was vortexed and then incubated in a water bath at 100 ˚C for 60 min to develop the malonaldehyde – TBA complex. The absorbance at 532 nm was measured after the solution had been cooled with cold tap water for 10 min. Values of TBARs were expressed as mg malonaldehyde per kg of sample. 1,1,3,3-tetraethoxypropane (TEP) was used for preparation of standard curve.

**Sensory evaluation. **Non-trained panelists (30) were recruited from the staff and postgraduate students of Department of Food Hygiene, Faculty of Veterinary Medicine, Urmia University, Urmia, Iran.^30^ A preparatory session was held prior to the testing, so that each panel members could thoroughly discuss and clarify each attribute in samples.

Testing was initiated after the panelists agreed on the specifications, in the Food Hygiene Laboratory of Urmia University, Urmia, Iran. The 9-point hedonic scale was carried out. During evaluation, the panelists were situated in private booths under incandescent light. Circle pieces were cut from the center of Lyoner slices and were served at room temperature.^31^ The sample presentation order was randomized for each panelist. Room temperature water was provided between samples to cleanse the palate. The attributes measured and their descriptors were as follows: for taste; acid taste, saltiness, and fatness (from imperceptible to extremely intense), for odor (from imperceptible to extremely intense); for color (from extremely light to extremely dark); and for texture: hardness (from extremely soft to extremely tough), juiciness (from extremely dry to extremely moist). At the end of the test, panelist gave a score for overall acceptability from 1 to 9. 

**Statistical analysis. **Conventional statistical methods were used to calculate means and standard deviations. Statistical analysis (ANOVA) was applied to the data to determine differences (*p* < 0.05). To discover whether there were significant differences between the levels of the main factor, contrasts (Tukey’s test) between means were made.^32^ The data were analyzed by SPSS (Version 16; SPSS Inc., Chicago, USA).

## Results

**Chemical characterization of CZEO. **As shown in Table 1, seventeen compounds of CZEO were identified, representing 93.15% of the total EO. The major compound groups were monoterpene hydrocarbons and phenolic compounds. Cinnamaldehyde (80.42%), α-Copaene (2.73%) and *trans*-Calamenene (2.16%) were the major chemical constituents of the oil. Other components analyzed in the oil were present in amounts less than 2.00%.

**Fig. 1:**
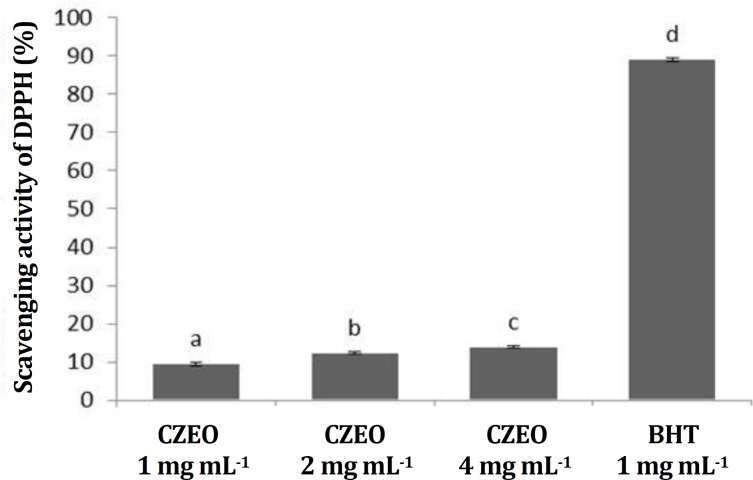
Free radical scavenging activity (%) of CZEO evaluated by DPPH assay. BHT (1 mg mL^-1^) was used as a positive control.

^abcd^ Different letters indicate significant differences among the DPPH scavenging activities (*p* < 0.05).

**Antioxidant activity. **The DPPH scavenging assay was used to indicate antioxidant activity of CZEO. This assay was based on the ability of DPPH, a stable free radical, to be quenched and thereby decolorize in the presence of antioxidants resulting in a reduction in absorbance values. The results showed that DPPH scavenging activity significantly was increased (*p* < 0.05) with increasing EO concentration as shown in Figure 1.

**Table 1:** Chemical constituents of *Cinnamomum zeylanicum* essential oil.

**Rt** * ** (Min)**	**Compound**	**KI** **	**Area (%)**
**13.94**	Styrene	NIST	0.26
**17.51**	Benzaldehyde	960	0.71
**28.14**	Benzenepropanal	NIST	0.24
**28.52**	Borneol	1169	0.31
**31.05**	(Z)Cinnamaldehyde	1219	1.68
**34.65**	(E)Cinnamaldehyde	1270	78.74
**38.59**	*α*-Copaene	1377	2.73
**41.25**	(E) Cinnamyl acetate	1446	0.58
**42.81**	gamma-Muurolene	1480	0.53
**42.94**	(ar-)Curcumene	1481	0.45
**43.82**	*α*-Muurolene	1500	1.62
**44.75**	*trans*-Calamenene	1529	2.16
**44.93**	2-Propenal,3-2 methoxyphenyl	1550	1.21
**49.55**	(epi-α) Cadinol	1640	0.87
**49.67**	*α*-Muurolol	1646	0.47
**50.79**	Cadalene	1677	0.25
**51**	(epi-α) Bisabolol	1685	0.29
**Total**		-	93.15

* Retention time

** Kovats indices calculated against n-alkanes on HP-5 column.

**Measuring composition of sausage samples.** The chemical composition of Lyoners is shown in Table 2. The moisture content fell in all the CZEO samples with the comparison to control sample, with no statistically significant differences (*p* > 0.05) between the CZEO 0.02% and CZEO 0.04% samples. The fat, protein and ash content did not show significant differences (*p* > 0.05) as a result of the addition of CZEO.

**Color** **measurement.** Table 3 shows color characteristics of sausages in accordance with the hunter system (L*, a*, b* color values), total color difference (ΔE) and whitish index (WI). In day 0 and 40, Lightness (L*) was significantly (*P* < 0.05) decreased by adding CZEO. No significant differences (*p* > 0.05) were found between the lightness of samples during 40 days storage in any samples. Redness (a*) and yellowness (b*) were significantly (*p* < 0.05) affected by the CZEO content. With the exception of first day of storage, these parameters were increased and decreased, respectively. Adding CZEO at day 0 and 40 resulted in a significant increase in total color difference (ΔE) and decrease in WI, (*p* < 0.05).

**pH and water activity measurement. **The pH values did not differ (*p *> 0.05) between the control and Lyoners containing CZEO, (Table 4). This parameter was significantly different in all of samples during the 40 days storage, except days 10 and 20 in samples containing CZEO.

The water activity content fell in Lyoners with CZEO compared with the control samples, with no significant differences (*p *> 0.05) between CZEO 0.02% and CZEO 0.04% samples at the first day of storage, (Table 4). But during storage time, water activity of control samples and the samples with CZEO decreased and increased respectively.

**Lipid oxidation.** Table 5 shows the effect of CZEO addition and storage time on the lipid oxidation of Lyoner. There was no significant difference between TBARs values of CZEO containing samples and control after 40 days of storage at 4 ˚C. All treated samples showed lower TBARs values than control and increasing of CZEO caused to decrease the TBARs values, but there was no statistically significance difference between them (*p* > 0.05). The TBARs values of all samples had an increasing trend during storage time, with statistically significant differences (*p* < 0.05) between days of storage.

**Sensory evaluation. **The results of sensory assessment of Lyoner samples are shown in Table 6. In general, samples with CZEO had the higher scores than control sample. Regarding to odor, the score of CZEO 0.04% sample was significantly higher than other samples (*p* < 0.05). The taste, color and overall acceptability scores of samples with CZEO were higher than control one, but there were no significant difference between them (*p* > 0.05). The texture of all samples was not significantly affected by addition CZEO (*p* > 0.05).

**Table 2:** Chemical composition of Lyoner formulated with CZEO (Mean ± SD).

**Formulation**	**Moisture (%)**	**Fat (%)**	**Protein (%)**	**Ash (%)**
**Control**	64.9 ± 0.02	19.57 ± 0.06	12.90 ± 0.02	2.50 ± 0.02
**CZEO (0.02%)**	62.93 ± 0.17*	19.61 ± 0.28	12.89 ± 0.02	2.45 ± 0.02
**CZEO (0.04%)**	63.04 ± 0.16*	19.57 ± 0.05	12.91 ± 0.14	2.50 ± 0.04

* indicates significant differences compared to control (*p* < 0.05).

**Table 3:** Color coordinates of Lyoners formulated with CZEO during 40 days of storage at 4˚C (Mean ± SD).

**Color coordinate **	**Sample**	**Storage Time (Day)**				
		**0**	**10**	**20**	**30**	**40**
**Lightness**	**Control**	67.52 ± 0.30^A^^a^	67.27 ± 0.28^A^^a^	67.09 ± 0.19^A^^a^	67.16 ± 0.74^A^^a^	67.09 ± 0.07^A^^a^
	**CZEO (0.02%)**	66.81 ± 0.18^B^^a^	66.80 ± 0.23^A^^a^	66.64 ± 0.31^A^^a^	66.65 ± 0.06^A^^a^	66.82 ± 0.08A^B^^a^
	**CZEO (0.04%)**	66.48 ± 0.12^B^^a^	66.68 ± 0.39^A^^a^	66.34 ± 0.14^A^^a^	66.54 ± 0.30^A^^a^	66.58 ± 0.03^B^^a^
**Redness**	**Control**	13.15 ± 0.01^A^^a^	12.86 ± 0.13^A^^bc^	12.60 ± 0.07^A^^c^	12.88 ± 0.14^A^^b^	12.65 ± 0.10A^bc^
	**CZEO (0.02%)**	13.26 ± 0.04^A^^a^	13.28 ± 0.06^B^^a^	13.25 ± 0.04^B^^a^	13.31 ± 0.04^B^^a^	13.31 ± 0.07^B^^a^
	**CZEO (0.04%)**	12.88 ± 0.15^B^^b^	13.14 ± 0.02^B^^a^	13.12 ± 0.07^C^^ab^	13.08 ± 0.04A^B^^ab^	13.03 ± 0.11^C^^ab^
**Yellowness**	**Control**	15.90 ± 0.03^A^^c^	16.41 ± 0.04^A^^b^	16.45 ± 0.02^A^^b^	16.43 ± 0.04^B^^b^	16.84 ± 0.13^A^^a^
	**CZEO (0.02%)**	16.19 ± 0.28^A^^c^	15.89 ± 0.02^B^^d^	16.32 ± 0.05^A^^c^	16.81 ± 0.08^A^^a^	16.56 ± 0.04^B^^b^
	**CZEO (0.04%)**	16.38 ± 0.07^A^^b^	16.34 ± 0.02^A^^b^	16.12 ± 0.01^B^^c^	16.35 ± 0.05^B^^b^	16.65 ± 0.02^AB^^a^
**Total color difference**	**Control**	32.01 ± 0.23^A^^a^	32.36 ± 0.17^A^^a^	32.42 ± 0.12^A^^a^	32.47 ± 0.47^A^^a^	32.66 ± 0.05^A^^a^
	**CZEO (0.02%)**	32.70 ± 0.25^B^^a^	32.60 ± 0.17^A^^a^	32.94 ± 0.19^A^^a^	33.22 ± 0.07^A^^a^	32.96 ± 0.01A^B^^a^
	**CZEO (0.04%)**	32.95 ± 0.04^B^^a^	32.88 ± 0.29^A^^a^	33.01 ± 0.14^A^^a^	32.97 ± 0.20^A^^a^	33.08 ± 0.3^B^^a^
**Whitish index**	**Control**	61.52 ± 0.26^A^^a^	61.19 ± 0.20^A^^a^	61.11 ± 0.14^A^^a^	61.08 ± 0.56^A^^a^	60.92 ± 0.02^A^^a^
	**CZEO (0.02%)**	60.76 ± 0.24^B^^a^	60.87 ± 0.19^A^^a^	60.57 ± 0.23^A^^a^	60.34 ± 0.07^A^^a^	60.60 ± 0.02^AB^^a^
	**CZEO (0.04%)**	60.53 ± 0.06B^C^^a^	60.63 ± 0.33^A^^a^	60.44 ± 0.15^A^^a^	60.52 ± 0.23^A^^a^	60.44 ± 0.02^B^^a^

ABC Values followed by the same capital letter within the same column are not significantly different (*p* > 0.05).

abc Values followed by the same small letter within the same row are not significantly different (*p* > 0.05).

**Table 4:** pH and water activity values of Lyoners formulated with CZEO stored at 4 ˚C (Mean ± SD).

**Parameter**	**Sample**	**Storage Time (Day)**				
		**0**	**10**	**20**	**30**	**40**
**pH**	**Control**	6.86 ± 0.00^A^^a^	6.75 ± 0.01^A^^b^	6.69 ± 0.01^A^^c^	6.37 ± 0.02^A^^d^	6.08 ± 0.01^A^^e^
	**CZEO 0.02%**	6.86 ± 0.00^A^^a^	6.76 ± 0.01^A^^b^	6.71 ± 0.02^A^^b^	6.38 ± 0.01^A^^c^	6.12 ± 0.01^A^^d^
	**CZEO 0.04%**	6.86 ± 0.00^A^^a^	6.77 ± 0.01^A^^b^	6.71 ± 0.00^A^^b^	6.39 ± 0.02^A^^c^	6.14 ± 0.00^A^^d^
**Water activity**	**Control**	0.98 ± 0.00^A^^a^	0.98 ± 0.00^A^^b^	0.98 ± 0.00^A^^a^	0.97 ± 0.00^A^^ab^	0.97 ± 0.00^A^^ab^
	**CZEO 0.02%**	0.97 ± 0.00^B^^b^	0.97 ± 0.00^A^^ab^	0.98 ± 0.00^A^^a^	0.98 ± 0.00^A^^ab^	0.98 ± 0.00^A^^ab^
	**CZEO 0.04%**	0.97 ± 0.00^B^^a^	0.97 ± 0.00^A^^a^	0.98 ± 0.00^A^^a^	0.98 ± 0.00^A^^a^	0.98 ± 0.00^A^^a^

AB Values followed by the same capital letter within the same column are not significantly different (*p* > 0.05).

abc Values followed by the same small letter within the same row are not significantly different (*p* > 0.05).

**Table 5:** Thiobarbituric acid reactive substances values (mg malonaldehyde per kg sample) of Lyoners formulated with CZEO, during 40 days of storage at 4 ˚C (Mean ± SD).

**Sample **	**Storage Time (Day)**				
	**0**	**10**	**20**	**30**	**40**
**Control**	1.32 ± 0.01^a^	1.75 ± 0.01^a^	2.28 ± 0.27^b^	2.93 ± 0.09^c^	3.57 ± 0.09^d^
**CZEO (0.02%)**	1.31 ± 0.01^a^	1.65 ± 0.16^b^	2.25 ± 0.07^c^	2.79 ± 0.02^d^	3.28 ± 0.09^e^
**CZEO (0.04%)**	1.32 ± 0.01^a^	1.62 ± 0.10^b^	2.24 ± 0.04^c^	2.74 ± 0.06^d^	3.25 ± 0.06^e^

There is no statistically significant difference among the values in the same column (*p* > 0.05).

abcd Values followed by the same small letter within the same row are not significantly different (*p* > 0.05).

**Table 6:** Sensory scores of Lyoners formulated with CZEO, during 40 days of storage at 4 ˚C (Mean ± SD).

**Sensory attributes**	**Sample**	**Storage Time (Days)**				
		**0**	**10**	**20**	**30**	**40**
**Taste**	**Control**	5.77 ± 1.30^Aa^	5.94 ± 1.24^Aa^	5.83 ± 1.20^Aa^	5.76 ± 2.04^Aa^	5.57 ± 2.00^Aa^
	**CZEO 0.02%**	6.10 ± 1.32^Aa^	6.42 ± 1.74^Aa^	6.15 ± 1.60^Aa^	6.35 ± 1.36^Aa^	6.33 ± 1.32^Aa^
	**CZEO 0.04%**	6.42 ± 1.34^Aa^	6.52 ± 2.01^Aa^	6.36 ± 1.46^Aa^	6.61 ± 1.61^Aa^	6.42 ± 1.64^Aa^
**Odor**	**Control**	5.77 ± 1.00^Aa^	5.94 ± 1.49^Aa^	5.83 ± 2.30^Aa^	5.76 ± 1.64^Aa^	5.57 ± 1.58^Aa^
	**CZEO 0.02%**	6.10 ± 1.16^Aa^	6.42 ± 1.10^Aa^	6.15 ± 1.24^Aa^	6.35 ± 1.62^Aa^	6.33 ± 1.63^Aa^
	**CZEO 0.04%**	6.42 ± 1.34^Ba^	6.52 ± 1.16^Ba^	6.36 ± 1.10^Ba^	6.61 ± 1.28^Ba^	6.42 ± 1.63^Ba^
**Color**	**Control**	6.33 ± 1.22^Aa^	6.50 ± 1.58^Aa^	4.27 ± 1.56^Ab^	5.66 ± 1.21^Aa^	5.89 ± 0.93^Aa^
	**CZEO 0.02%**	6.47 ± 1.38^Aa^	6.34 ± 1.37^Aa^	5.05 ± 1.61^Ab^	5.88 ± 1.43^Aab^	6.10 ± 1.14^Aab^
	**CZEO 0.04%**	6.52 ± 0.84^Aa^	6.26 ± 1.62^Aa^	4.94 ± 1.43^Ab^	6.00 ± 1.63^Aab^	6.00 ± 1.49^Aab^
**Texture**	**Control**	7.00 ± 1.32^Aa^	6.76 ± 1.43^Aab^	6.00 ± 1.81^Aab^	5.89 ± 1.38^Ab^	5.50 ± 1.94^Aab^
	**CZEO 0.02%**	6.21 ± 1.65^Aab^	6.89 ± 1.44^Aa^	6.72 ± 1.44^Aa^	5.78 ± 1.52^Ab^	5.00 ± 2.01^Aab^
	**CZEO 0.04%**	6.57 ± 1.38^Aa^	6.89 ± 1.66^Aa^	6.73 ± 1.69^Aa^	6.05 ± 1.80^Ab^	4.63 ± 1.31^Aab^
**Overall acceptability**	**Control**	6.83 ± 1.20^Aa^	6.68 ± 0.47^Aa^	5.55 ± 1.09^Ab^	6.11 ± 1.77^Aab^	5.68 ± 0.47^Ab^
	**CZEO 0.02%**	7.13 ± 0.84^Aa^	6.26 ± 1.09^Ab^	6.15 ± 0.89^Aab^	6.31 ± 0.94^Ab^	6.15 ± 0.68^Ab^
	**CZEO 0.04%**	7.55 ± 0.55^Aa^	6.31 ± 1.33^Ab^	6.10 ± 1.93^Ab^	6.94 ± 1.39^Aab^	6.42 ± 0.83^Ab^

ab Values followed by the same small letter within the same row are not significantly different (*p* > 0.05).

AB Values followed by the same capital letter within the same column are not significantly different (*p* > 0.05).

## Discussion

Results of GC-MS analysis showed that CZEO is rich in monoterpene phenols, especially cinnamaldehyde that has antioxidant properties.^19^^,^^20^^,^^33^ Other researchers have also shown cinnamaldehyde to be the major component in CZEO. El-Baroty *et al.* reported that the trans-cinnamaldehyde with percentages of 45.13% is the main component in CZEO.^34^^-^^37^ Moreover, a number of studies have been published on the composition of the essential oil from *C. zeylanicum* bark. Similarly, main component of cinnamon bark oil was cinnamaldehyde ranging 44.20% to 97.70%.^36^^,^^38^^-^^43^ Previous studies have shown variations in essential oil composition that may be due to species difference, region climatic condition, stage of maturity, distillation conditions and other factors.^34^

DPPH scavenging assay was used to indicate antioxidant activity of CZEO. The DPPH scavenging activity was increased with increasing the concentration of EO (*p* < 0.05). CZEO at 4 mg mL^-1^ showed the highest percentage DPPH radical scavenging activity (13.92%). Our finding is in agreement with that of Aliakbarlu *et al.* who reported a low radical scavenging activity (13.02%) for CZEO.^24^ But in other study, Schmidt *et al.* reported that CZEO demonstrated the highest inhibitory activity compared to the other natural and chemical antioxidants.^44^ There is a direct correlation between the free radical scavenging and/or antioxidant activity of *C. zeylanicum* extract and concentration.^20^ Also, a linear trend was observed between polyphenolic concentration and radical scavenging activity of different plants.^45^^,^^46^

Lightness in food is related with many factors, including the concentration and type of pigments present,^47^ water content,^48^ and essential oils content and type.^49^ The results showed that by increasing CZEO concentration, L* values were decreased and color of samples tended to darken. 

With the exception of first day of storage, the red–green coordinate (a*) of CZEO samples significantly increased with respect to the control (*p* < 0.05). This coordinate is affected by the structural integrity of the food, the pigment content and disposition (water or lipid-soluble) and surface water availability.^50^ This parameter, whether from a positive (red) or negative (green) point of view, could have a linear relationship with the concentration of pigment.^7^

At the first day of storage, redness (a*) and yellowness (b*) of samples were decreased and increased, respectively. The decreased a* values and increased b* values, with or without L* changes, are associated with the fading of the cured color.^51^ The fading that resulted from adding high concentrations of EO can be explained by a possible interaction between nitrite and chemical components present in the aromatic fraction EO, making NO_2_ unavailable to combine with myoglobin to produce the characteristic red color.^52^ This finding is in agreement with Lindahl *et al*. who found that the pigment content and the myoglobin form were the most important factors in the variation in a* value.^47^

During the 40 days of storage, total color differences (ΔE) of CZEO samples are upper than control samples. This was not in agreement with findings of Estévez *et al*. who observed that higher levels of rosemary essential oil (0.03% and 0.06%) caused significant reduction on ΔE in comparison with control samples.^53^

Regarding to pH values, CZEO did not show statistically significant effect (*p* > 0.05) on pH in each day of storage days, while time was the most influential factor in this respect. In the control, CZEO 0.02% and CZEO 0.04% samples, the pH significantly decreased from 6.86 at day 0 to 6.08, 6.12 and 6.14, respectively, at day 40 (*p* < 0.05).

This phenomenon may have been due to the fall in pH coincided with the more growth of lactic acid bacteria in control sample in compare with other samples, which would lead to more lactic acid production. ^54^

Table 4 shows the water activity in Lyoners. At days 0 and 10, CZEO samples have lower water activity content than control sample. This reduction in water activity content at the first days of storage, is probably due to the fact that essential oils have a high water holding capacity.^55^

The results of TBARs measurements are shown in Table 5. All samples had lower TBARs values than control sample and adding CZEO decreased the lipid oxidation of samples. The antioxidant activity of spice essential oils in general and in rosemary and thyme in particular, is accepted.^46^^,^^56^ Such activity is basically due to the composition of essential oils: mainly to flavonoids and phenolic compounds. Flavonoids act as antioxidants because their structural features like scavenging lipid peroxy radicals by donating hydrogen and become more stable phenoxy radicals.^57^ Spice essential oils with anti-oxidant activities may also interfere with the propagation reactions ^58^ besides inhibiting the enzymatic systems involved in initiation reactions ^59^ or they can act as the hydrogen donor, scavengers of free radicals, metallic ion chelation or even acting as substrate of radicals such as superoxide or hydroxyl.^60^

According to the results of sensory evaluation, samples with CZEO had the higher scores than control ones in all of sensory attributes. The addition of natural essential oils have been reported to enhance texture characteristics of emulsion type meat products by reducing hardness, adhesiveness, gumminess and chewiness.^44^^,^^53^ But the results of the present study showed that the texture of samples was not significantly affected by adding CZEO (*p* > 0.05). The samples with CZEO showed the highest values of color intensity. The panelists clearly detected the difference of color produced in the samples. As regards lightness (L*), differences arose between the appreciation of this property as measured by panelists and instrumental means. The panelists detecting better color in CZEO samples, while the instrumental measurement of L* showed lower values for these samples. In agreement with our findings, it has been reported that overall acceptability of fish fillet sample coated with solution containing *C. zeylanicum* essential oil was higher than control sample at 16^th^ day.^61^

In conclusion, the addition of spice essential oils such as cinnamon seems to be a technologically viable alternative for elaborating cooked meat products because the ”natural” image of the products is improved. However the new and beneficial aspects of innovative products must be properly communicated to the consumer in an easily comprehensible manner. In the case of Lyoner, CZEO improve acceptance and have desirable effects as regards oxidative stability. The addition of CZEO alone and in combination with other preservative agents and methods (such as other plant-originated antioxidant agents or vacuum packaging) should be considered as a good method to improve chemical characteristics of Lyoner-type sausage.
